# Alveolar repair following LPS-induced injury requires cell-ECM interactions

**DOI:** 10.1172/jci.insight.167211

**Published:** 2023-07-24

**Authors:** Jennifer M.S. Sucre, Fabian Bock, Nicholas M. Negretti, John T. Benjamin, Peter M. Gulleman, Xinyu Dong, Kimberly T. Ferguson, Christopher S. Jetter, Wei Han, Yang Liu, Seunghyi Kook, Jason J. Gokey, Susan H. Guttentag, Jonathan A. Kropski, Timothy S. Blackwell, Roy Zent, Erin J. Plosa

**Affiliations:** 1 Department of Pediatrics, Division of Neonatology;; 2 Department of Cell and Developmental Biology;; 3 Department of Medicine, Division of Nephrology and Hypertension; and; 4 Department of Medicine, Division of Allergy, Pulmonary, and Critical Care Medicine, Vanderbilt University Medical Center, Nashville, Tennessee, USA.; 5 Nashville Veterans Affairs Medical Center, Nashville, Tennessee, USA.

**Keywords:** Cell Biology, Pulmonology, Integrins, Respiration

## Abstract

During alveolar repair, alveolar type 2 (AT2) epithelial cell progenitors rapidly proliferate and differentiate into flat AT1 epithelial cells. Failure of normal alveolar repair mechanisms can lead to loss of alveolar structure (emphysema) or development of fibrosis, depending on the type and severity of injury. To test if β_1_-containing integrins are required during repair following acute injury, we administered *E*. *coli* lipopolysaccharide (LPS) by intratracheal injection to mice with a postdevelopmental deletion of β_1_ integrin in AT2 cells. While control mice recovered from LPS injury without structural abnormalities, β_1_-deficient mice had more severe inflammation and developed emphysema. In addition, recovering alveoli were repopulated with an abundance of rounded epithelial cells coexpressing AT2 epithelial, AT1 epithelial, and mixed intermediate cell state markers, with few mature type 1 cells. AT2 cells deficient in β_1_ showed persistently increased proliferation after injury, which was blocked by inhibiting NF-κB activation in these cells. Lineage tracing experiments revealed that β_1_-deficient AT2 cells failed to differentiate into mature AT1 epithelial cells. Together, these findings demonstrate that functional alveolar repair after injury with terminal alveolar epithelial differentiation requires β_1_-containing integrins.

## Introduction

During lung development, primordial epithelial cells proliferate, migrate, and change phenotypic identity with precise timing, anchored by signals from the basement membrane (BM), a specialized extracellular matrix (ECM) structure, which is precisely remodeled at specific developmental checkpoints (1). Once lung development is complete, targeted replacement of epithelial cells and slow turnover of the alveolar BM protect the alveolar structure during homeostasis in the mature lung. However, epithelial cells lose capacity for efficient proliferation and differentiation as the BM ages (2), increasing susceptibility to chronic lung diseases over time. In contrast to temporally precise development and the relatively quiescent adult lung, repair of acute lung injury must occur rapidly to restore a gas-exchanging epithelium to maintain survival. The immediacy of repair results in epithelial proliferation and differentiation that occurs in bulk. For a wild-type mouse, the mild lung injury induced by a single dose of intratracheal lipopolysaccharide (LPS) is easily recoverable in days, overcoming inflammation-derived proteolytic damage to the BM that occurs within hours (3, 4). LPS, as a model of acute inflammatory lung injury, tests the regenerative potential of the alveolus, exposing impairments in epithelium-ECM interactions that could exacerbate lung injury or predispose to accelerated aging.

Integrins are heterodimeric transmembrane protein receptors composed of an α and β subunit that bind ECM ligands. Integrins provide physical connections between cells and the ECM, and they propagate signaling to and from the surrounding matrix (5–7). Of the 24 integrin heterodimers, 12 contain the β_1_ subunit, and many of the 12 β_1_-containing integrins are present in epithelial tissues. Integrin function is dependent upon developmental and microenvironmental context, a concept consistent with our previous work. We previously reported that epithelial β_1_ integrins are required for normal lung development, and in their absence airway branching and alveolarization are impaired and associated with incomplete epithelial differentiation in the later stages of lung development (8). However, in the homeostatic postdevelopmental adult alveolus, β_1_ integrins are completely dispensable for alveolar epithelial differentiation (9). Function of the alveolar stem cell niche during repair depends on the rapid proliferation of distal epithelial progenitors, followed by en masse differentiation. The degree of differentiation in this context is profound, and any inefficiencies in this process will create structural vulnerability and abnormal repair. Whether or not β_1_-containing integrins are required for the epithelial repair cycle in the acutely injured adult alveolus is unknown.

The adult alveolar BM is primarily composed of collagen IV and laminin and facilitates gas exchange between the airspace and the capillary network. The vast majority of the BM is covered with very thin and outspread type 1 alveolar epithelial cells (AT1 cells), interspersed with single cuboidal type 2 alveolar epithelial cells (AT2 cells). Each cell type has essential functions: AT2 cells secrete surfactant to reduce alveolar surface tension and function in an immunoregulatory role, while the thin AT1 cells allow gas exchange, maintain water homeostasis, and produce BM components (10). Following alveolar injury in both human lung disease and murine models, multiple types of epithelial progenitors restore epithelial coverage of the BM (11, 12). The most proximate and immediate epithelial progenitors for alveolar repair are AT2 cells themselves, a subset of which proliferate upon alveolar injury (13–15). Overproliferation of AT2 cells is restrained by various proteins, including the tight junction protein claudin-18 (16). After proliferation, the AT2 progenitors attain an AT2-to-AT1 transitional cell state with characteristic expression of genes such as *Krt8, Hbegf,* or *Areg* (11, 17, 18), on their way to becoming replacement AT1 cells. Less is understood about the molecular mechanisms that govern the final point of transition in alveolar repair, the acquisition of the AT1 transcriptional phenotype, and the full extension of the AT1 outspread cell shape.

Here, we compared distal lung epithelium restoration after acute lung injury induced by intratracheal LPS administration in normal adult mice with those carrying a β_1_ integrin inactivation targeted to surfactant protein C–expressing (SP-C–expressing) AT2 cells. From this modest acute lung injury, β_1_-deficient mice exhibited increased inflammation and had exacerbated lung injury, followed by emphysema. Impaired alveolar repair mechanisms in injured β_1_-deficient mice resulted in excessive AT2 proliferation, progressive accumulation of AT2/AT1 intermediate cells, and large-scale failure of AT1 differentiation. In these studies, the degree of cell shape block in β_1_-deficient mice demonstrated that AT2-to-AT1 differentiation is tightly integrated with cell shape change. Further, we showed that epithelial differentiation during repair bears a particular requirement for β_1_ integrin–mediated cell-ECM interactions, more than AT2-to-AT1 differentiation in other contexts. Unlike the assumption that AT2-to-AT1 epithelial differentiation processes employ similar basic mechanistic pathways no matter the context, our data suggest a different paradigm for adult injury repair wherein the rapidity with which differentiation occurs and/or the condition of the ECM leads to distinct differentiation mechanisms.

## Results

### Deletion of β_1_ integrin in AT2 cells increases susceptibility to injury and abnormal repair.

**Figure 1:**
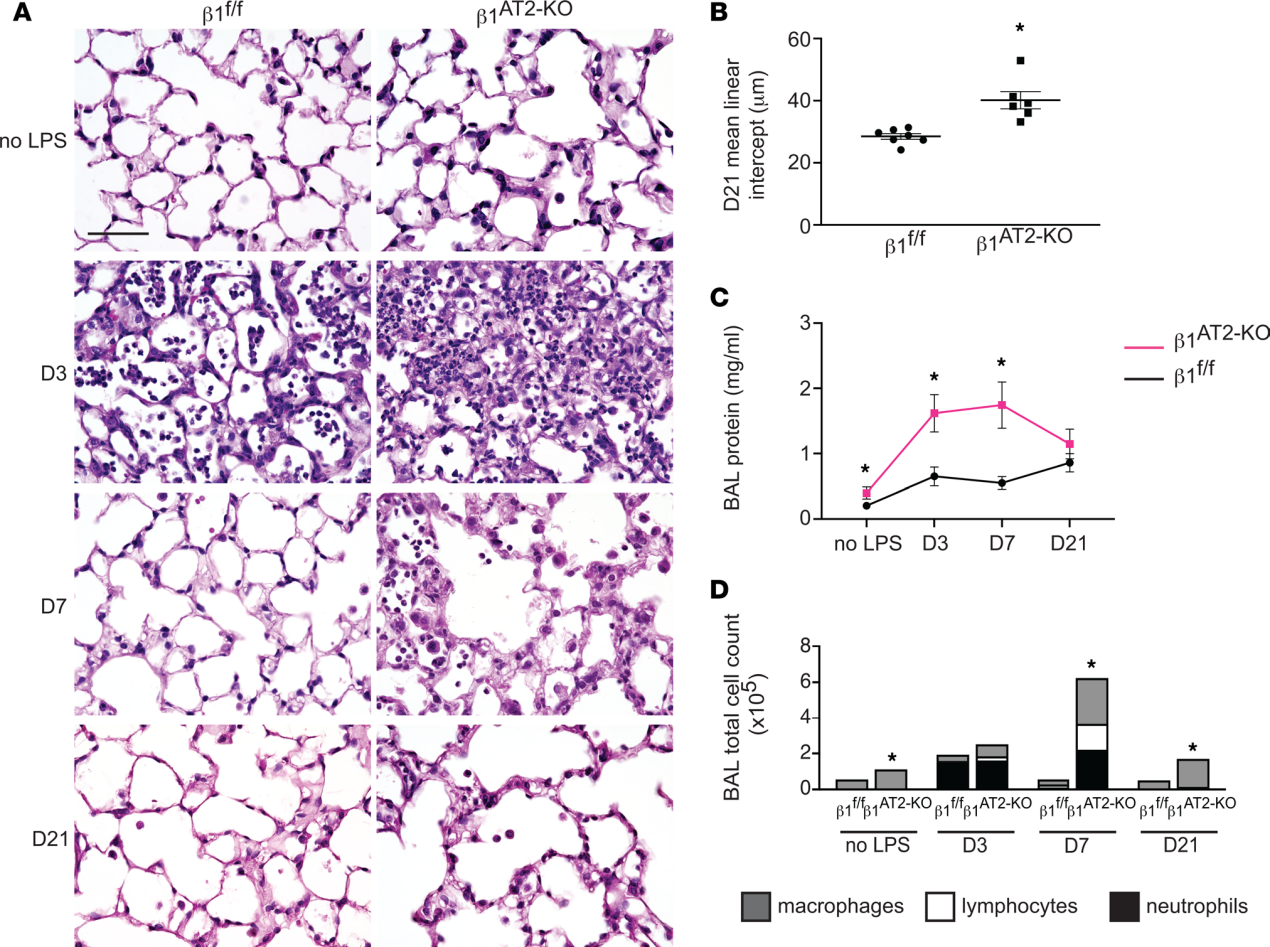
Deletion of β_1_ integrin in AT2 cells results in increased inflammation, abnormal repair, and decreased survival after LPS-induced injury. (**A**) Representative images of lung histology demonstrate increased edema at 3 and 7 days postinjury in β_1_^AT2-KO^ lungs compared with β_1_^fl/fl^ lungs, as well as persistent inflammation and emphysematous remodeling in β_1_^AT2-KO^ lungs by 21 days post-LPS. D3, D7, and D21 refer to 3, 7, and 21 days after LPS injury, respectively. (**B**) Mean linear intercept quantified emphysematous alveolar remodeling at 21 days after LPS, 28.5 ± 0.9 μm in β_1_^fl/fl^ lungs versus 40.2 ± 2.8 μm in β_1_^AT2-KO^ lungs (*n* = 6–7 mice/group, *P* = 0.0014 by 2-tailed *t* test). (**C**) Bicinchoninic acid (BCA) protein assay quantified increased bronchoalveolar lavage (BAL) fluid protein in uninjured β_1_^AT2-KO^ lungs and at 3 and 7 days post-LPS injury in β_1_^AT2-KO^ lungs compared with β_1_^fl/fl^ lungs at the same time points (*n* = 6–14 mice/group, 2-tailed *t* test comparing genotypes at each time point, *P* = 0.0485 for uninjured mice; *P* = 0.0036 at D3; *P* = 0.005 at D7; *P* = 0.2628 at D21). (**D**) BAL cell counts are significantly increased in β_1_^AT2-KO^ lungs compared with β_1_^fl/fl^ littermates in uninjured mice and at 7 and 21 days post-LPS. Peak inflammation is present at 7 days in β_1_^AT2-KO^ lungs, 55,663 ± 3,306 cells/mL in β_1_^fl/fl^ BAL fluid versus 624,000 ± 118,753 cells/mL in β_1_^AT2-KO^ BAL (*n* = 6–26 mice/group, 2-tailed *t* test comparing genotypes at each time point, *P* = 0.0002 for uninjured mice; *P* = 0.0730 at D3; *P* = 0.0007 at D7; *P* < 0.0001 at D21). Total numbers of BAL fluid macrophages are significantly increased in uninjured β_1_^AT2-KO^ mice and at D7 and D21; lymphocytes and neutrophils are significantly increased in β_1_^AT2-KO^ BAL at D7 only. Scale bar = 50 μm for **A**. * *P* < 0.05.

To determine the role of epithelial β_1_ integrin in alveolar repair after injury, we generated mice with a β_1_ integrin deletion in AT2 cells in the adult lung utilizing the SP-C rtTA TetO-Cre doxycycline-inducible system (9, 19) and floxed β_1_ integrin (β_1_^fl/fl^), hereafter referred to as β_1_^AT2-KO^ mice. We previously reported the efficient deletion of β_1_ integrin in AT2 cells (then termed β_1_^rtTA^ mice). These mice exhibited grossly normal airspace size at 3 months of age but also increased epithelial inflammation and tight junction defects paired with modest intraseptal edema (9). We confirmed sustained β_1_ integrin expression in β_1_^fl/fl^ littermate control mice and normal histology in SP-C rtTA TetO-Cre β_1_^fl/fl^ mice not receiving doxycycline at 3 months of age (9, 19). While doxycycline toxicity has been reported with embryonic administration (20), we observed no overt histologic or biological toxicity from the rtTA system or from doxycycline itself in this postdevelopmental model (9). After 1 month off doxycycline to minimize side effects from possible doxycycline tissue storage, we challenged β_1_^AT2-KO^ and littermate β_1_^fl/fl^ controls with a single intratracheal (IT) dose of LPS at 3 months of age (Figure 1A). β_1_^AT2-KO^ mice exhibited decreased survival compared with β_1_^fl/fl^ control mice (44% survival at 21 days for β_1_^AT2-KO^ versus 93% for β_1_^fl/fl^, *n* = 15 β_1_^AT2-KO^ and 14 β_1_^fl/fl^ mice, *P* = 0.0051, Supplemental Figure 1A; supplemental material available online with this article; https://doi.org/10.1172/jci.insight.167211DS1), with 100% survival for both PBS-treated β_1_^AT2-KO^ and β_1_^fl/fl^ mice (data not shown). Three days postinjury, histological examination revealed an expected increase in inflammatory cells in both LPS-treated β_1_^AT2-KO^ and control β_1_^fl/fl^ lungs and markedly increased edema in β_1_^AT2-KO^ lungs. Whereas LPS-treated β_1_^fl/fl^ lungs returned to a normal histological appearance by day 7, edema and inflammation remained prominent in β_1_^AT2-KO^ lungs, consistent with an exacerbation of acute lung injury. Failed alveolar repair was evident at 21 days in β_1_^AT2-KO^ mice, whose lungs exhibited a marked increase in airspace size and septal destruction. Emphysema was quantified by increased mean linear intercept (28.5 ± 0.9 μm in β_1_^fl/fl^ lungs vs. 40.2 ± 2.8 μm in β_1_^AT2-KO^ lungs) (Figure 1B). To evaluate alveolar barrier function, we measured bronchoalveolar lavage (BAL) fluid protein levels at all time points (Figure 1C). β_1_^AT2-KO^ BAL fluid contained increased protein in the unchallenged state, as well as at 3 and 7 days post-LPS compared with control β_1_^fl/fl^ BAL fluid. By 21 days postinjury, protein levels were similar in both mouse strains, indicating restoration of alveolar barrier function in β_1_^AT2-KO^ lungs.

We collected BAL fluid to characterize the inflammatory response to LPS and found no significant differences in immune/inflammatory cells between β_1_^AT2-KO^ and β_1_^fl/fl^ lungs at 3 days, during the expected peak of inflammation following LPS (21) (Figure 1D). However, in β_1_^AT2-KO^ mice, there was a delayed inflammatory peak at 7 days postinjury characterized by persistently increased neutrophils in BAL fluid from β_1_^AT2-KO^ lungs (Supplemental Figure 1B). Although the BAL cell count decreased toward baseline at 21 days in β_1_^AT2-KO^ lungs, increased numbers of macrophages remained. These findings were verified by quantification of immunostaining for the macrophage marker CD68 (Supplemental Figure 1, C and D). Since chronic inflammation predisposes to emphysema, we tested if the exaggerated acute inflammatory response contributed to airspace expansion in LPS-treated β_1_^AT2-KO^ lungs. We delayed doxycycline administration for 5 days after IT LPS dosing, thereby inducing β_1_ integrin deletion after acute inflammation. Lungs were harvested 21 days after LPS treatment (and 16 days after the initiation of doxycycline). On histological examination, β_1_^AT2-KO^ lungs retained emphysematous structural deficits, quantified by an increased mean linear intercept (Supplemental Figure 1, E and F).

We next investigated whether LPS-treated β_1_^AT2-KO^ mice exhibited fibrosis, an additional manifestation of abnormal alveolar repair apart from emphysema. By Masson’s trichrome staining, β_1_^AT2-KO^ and β_1_^fl/fl^ lungs did not exhibit gross differences in fibrillar collagen deposition (Supplemental Figure 2A). The alveolar BM also appeared similarly composed between genotypes, with comparable pan-laminin localization in the alveolar septum 21 days after LPS treatment (Supplemental Figure 2B). As loss of 1 integrin may result in the upregulation of alternative integrin subunits, we examined LPS-treated β_1_^AT2-KO^ and β_1_^fl/fl^ lungs for differences in Arg-Gly-Asp–binding (RGD-binding) integrins. In this subgroup of integrins, heterodimers composed of varied β subunits bind ECM proteins containing the highly conserved RGD motif (22). We first verified continued absence of β_1_ at 21 days after LPS in β_1_^AT2-KO^ AT2 cells. Integrin α_5_, which partners only with β_1_ to form a fibronectin receptor, was also decreased throughout injured β_1_^AT2-KO^ lungs compared with β_1_^fl/fl^ lungs (Supplemental Figure 3A). In contrast to α_5_β_1_, the α_v_ RGD-binding integrins were increased (Supplemental Figure 3, B–E), particularly the β_5_ and β_8_ subunits, suggesting compensation by α_v_β_5_ and α_v_β_8_ without overt fibrosis in the injured β_1_^AT2-KO^ lung. Taken together, these findings indicate that loss of β_1_-containing epithelial integrins drives increased LPS-induced inflammation and lung injury, followed by emphysema and abnormal alveolar repair.

### AT2 cells deficient in β_1_ increase in number post-LPS injury.

**Figure 2:**
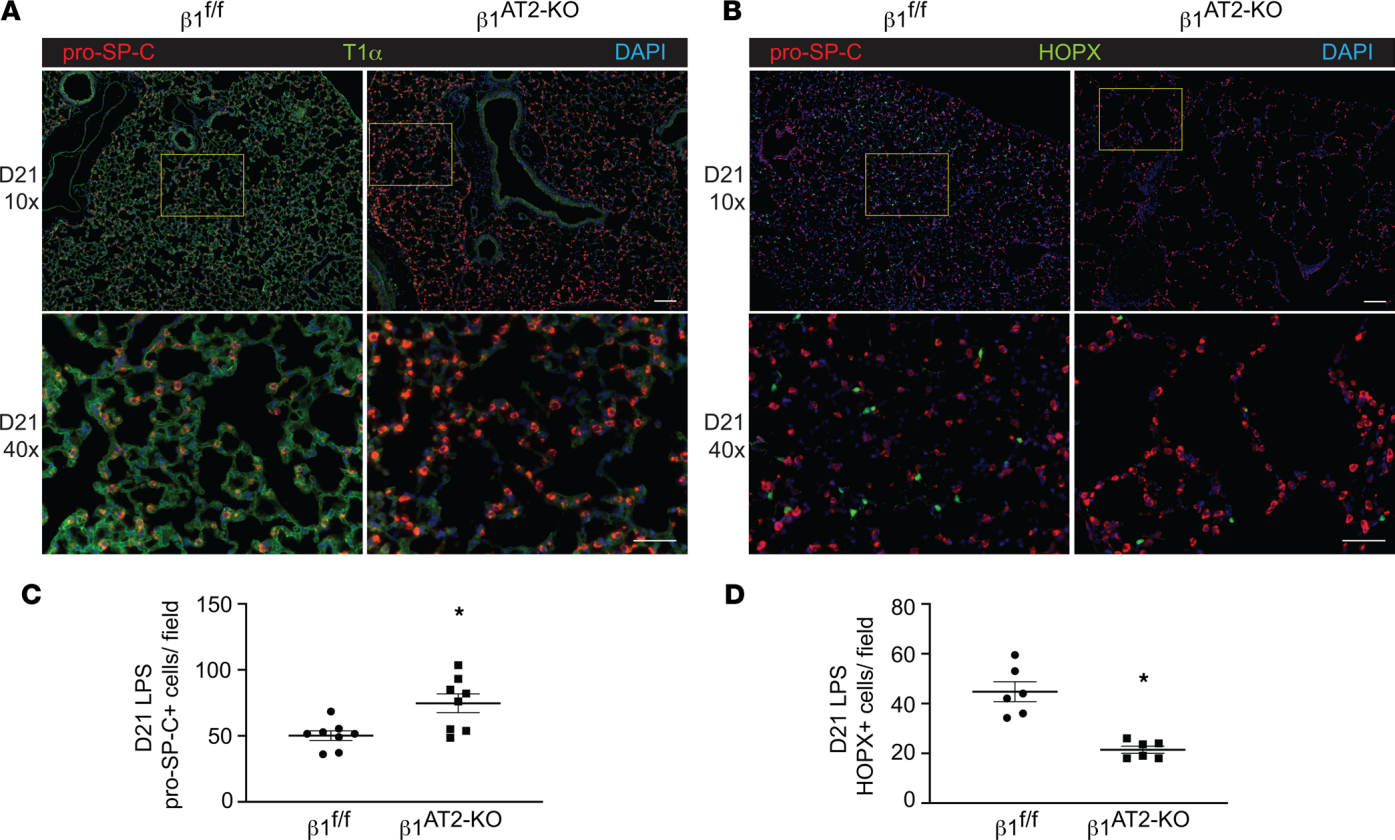
Lungs deficient in β_1_have increased AT2 cell number and decreased AT1 cells at 21 days after LPS injury. (**A**) Representative low-power (original magnification, 10×) and inset high-power images (original magnification, 40×, denoted by yellow boxes) of β_1_^fl/fl^ and β_1_^AT2-KO^ lungs 21 days after LPS (D21), immunostained for AT2 marker pro–SP-C (red) and AT1 marker T1α (green). (**B**) Representative images of D21 β_1_^fl/fl^ and β_1_^AT2-KO^ lungs immunostained for pro–SP-C (red) with the AT1 marker Hopx (green), insets as in **A**. (**C**) Quantification of the number of pro–SP-C^+^ AT2 cells per field (*n* = 8 mice/group, 10 original magnification, 40× sections/mouse; *P* = 0.0086). (**D**) Hopx^+^ cells per field (*n* = 6 mice/group, 6 original magnification, 20×, sections/mouse; *P* = 0.0003). * *P* < 0.05. Scale bar = 100 μm for 10× in **A** and **B**; scale bar = 50 μm for 40× in **A** and **B**. Two-tailed *t* test was used to compare genotypes in **C** and **D**.

**Figure 3:**
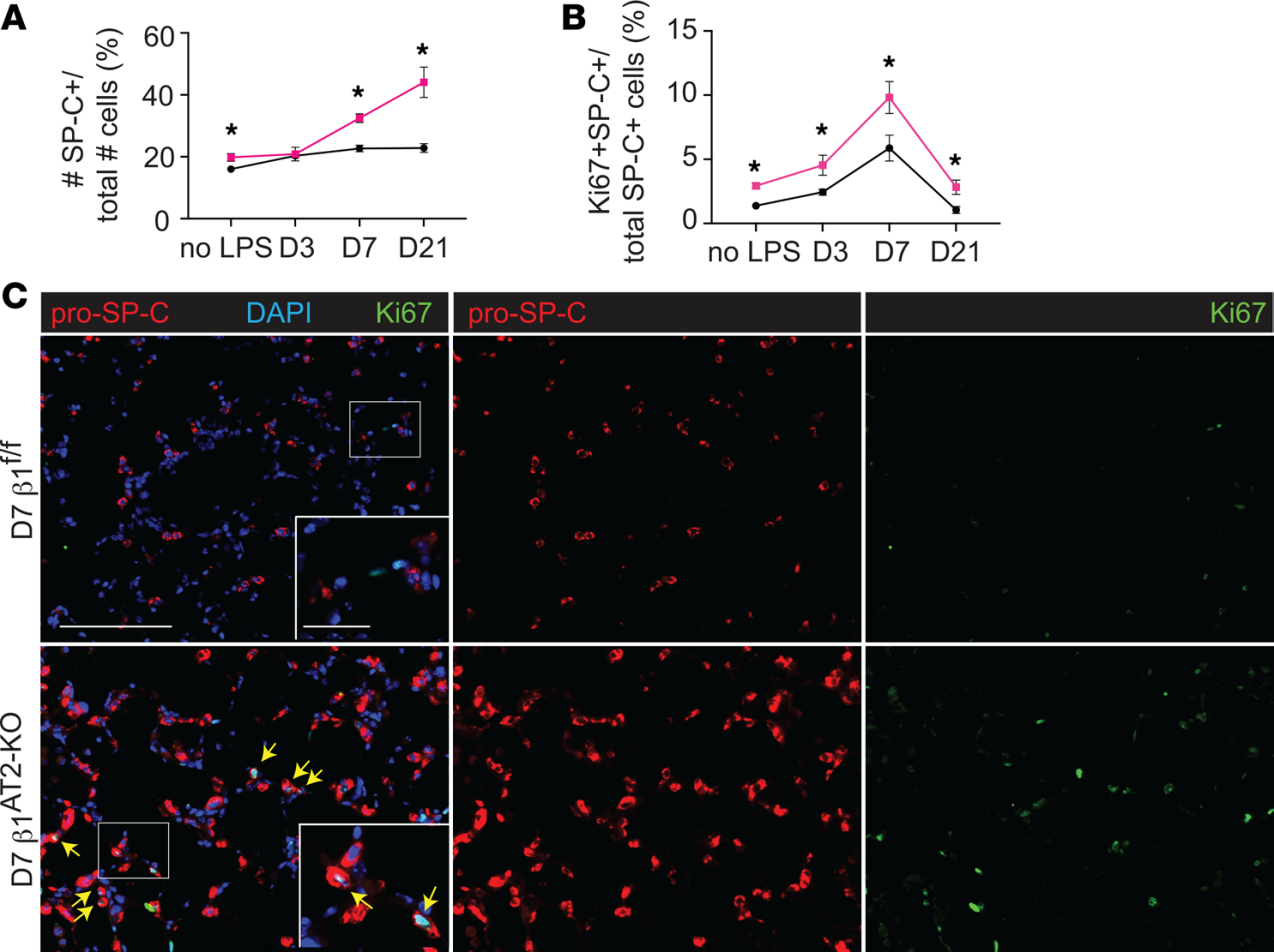
β_1_^AT2-KO^ mice have increased AT2 proliferation during alveolar repair. (**A**) Quantification of pro–SP-C^+^ cells per total cells (*n* = 6–8 mice/group; 5 sections per mouse; *P* = 0.0247 for uninjured (no LPS) mice; *P* = 0.8220 at D3; *P* = 0.0001 at D7; *P* = 0.0009 at D21). (**B**) Quantification of proliferating AT2 cells by percentage of total pro–SP-C^+^ AT2s (*n* = 6–8 mice/group, 10 sections/mouse; *P* = 0.0003 for uninjured mice; *P* = 0.0311 at D3; *P* = 0.0310 at D7; *P* = 0.0128 at D21). (**C**) Immunodetection of the proliferation marker Ki-67 (green) and pro–SP-C (red) shows peak AT2 proliferation in β_1_^AT2-KO^ lungs at day 7 (arrows) as merged or single-channel panels. Two-tailed *t* test was used to compare genotypes at each time point for **A** and **B**. * *P* < 0.05. Scale bar = 200 μm for low-power image in **C**, 50 μm for inset in **C**.

**Figure 4:**
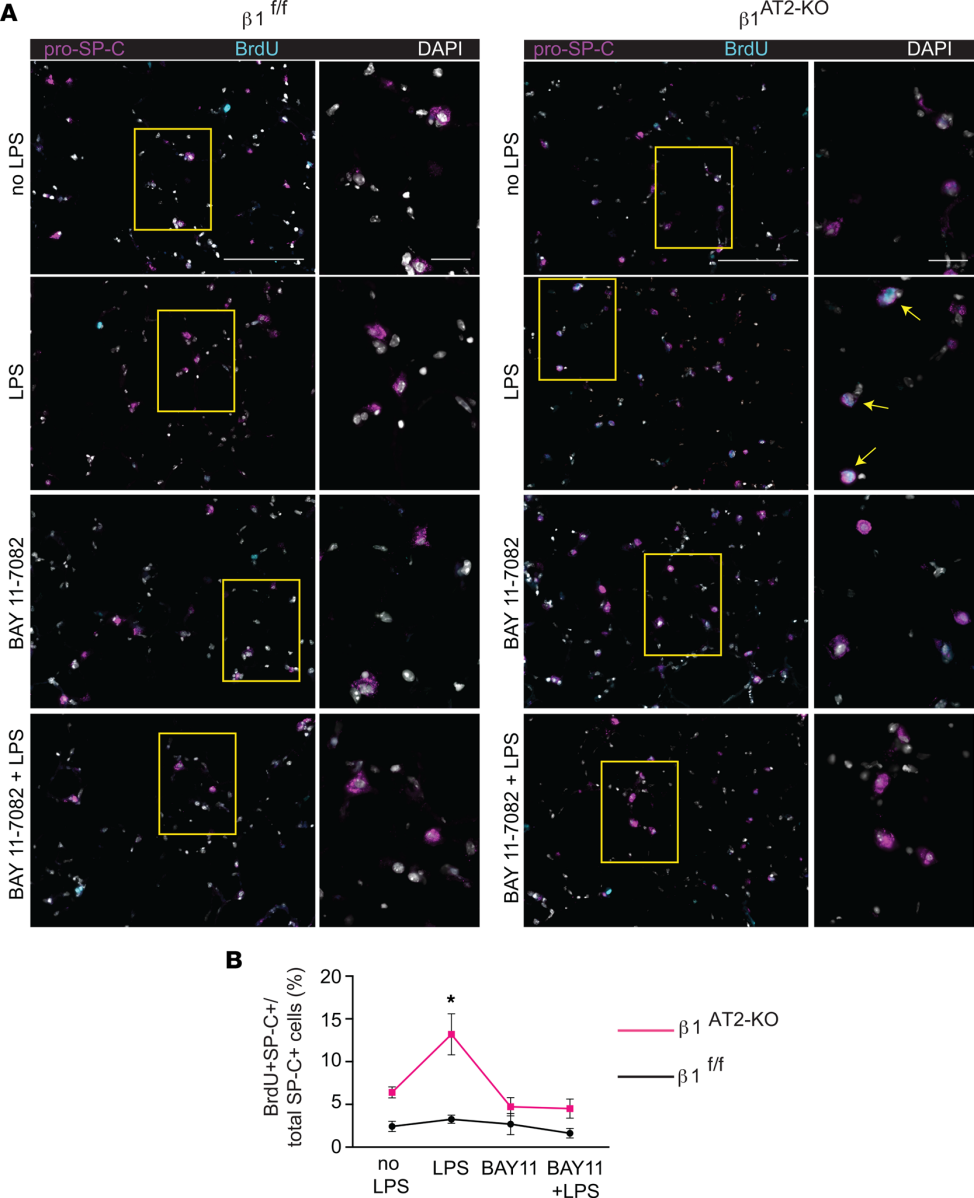
AT2 proliferation is NF-κB dependent in LPS-treated β_1_^AT2-KO^ lungs. (**A**) Representative images of BrdU-incorporated precision-cut lung slices (PCLS) treated with LPS and/or NF-κB inhibitor BAY 11-7082 for 48 hours. Slices were immunostained for BrdU (cyan) and pro–SP-C (magenta) with DAPI nuclear marker (white). (**B**) Quantification of proliferating AT2 cells by percentage of total AT2 cells (BrdU^+^pro–SP-C^+^ over total number of SP-C^+^ cells) by condition as indicated (*n* = 6–8 mice/group, 1 slice per mouse per condition, imaged and quantified 10 original magnification, 40× sections/mouse per condition, data from 5 separate experiments; *P* = 0.0010, *F* value = 6.3 for treatment variation; *P* < 0.0001, *F* value = 26.1 for genotype variation). Two-way ANOVA was used to compare treatment conditions and genotype in **B**. * *P* < 0.05. Scale bar = 100 μm for low-power images in **A**, 50 μm for inset in **A**.

To determine how β_1_ deficiency in AT2 cells alters alveolar repair, we performed an in-depth histological and transcriptomic examination of alveolar epithelial cells in β_1_^AT2-KO^ and β_1_^fl/fl^ lungs. Lung sections immunostained for the AT2 marker pro–SP-C and AT1 marker T1α demonstrated accumulation of pro–SP-C^+^ AT2 cells in β_1_^AT2-KO^ lungs by day 7 following LPS injury (Supplemental Figure 4). By day 21, β_1_^AT2-KO^ lungs contained increased numbers of pro–SP-C^+^ AT2 cells throughout the lung parenchyma (Figure 2, A–C). Moreover, severely injured areas exhibited alveolar septa lined by abundant pro–SP-C^+^ AT2 cells. The increase in AT2 cell number was accompanied by decreased expression of the AT1 markers T1α and HOP homeobox (Hopx) in β_1_^AT2-KO^ lungs (Figure 2D), suggesting loss of AT1 cells and large expansion of the AT2 population. We captured the increased imbalance between AT2 and AT1 cells in β_1_^AT2-KO^ lungs throughout repair by calculating the number of pro–SP-C^+^ cells as a percentage of total cells (Figure 3A). Whereas the percentage of AT2 cells remained stable from day 3 into late repair in the β_1_^fl/fl^ lungs, the percentage of pro–SP-C^+^ AT2 cells progressively increased throughout later repair in β_1_^AT2-KO^ lungs. The increased AT2 number in β_1_^AT2-KO^ lungs prompted an examination of proliferation and apoptosis. We assessed AT2 proliferation by co-immunostaining lung sections for the proliferation marker Ki-67 and pro–SP-C. β_1_^AT2-KO^ lungs exhibited increased proliferation in pro–SP-C^+^ AT2 cells at all sampled time points from uninjured lungs to day 21 postinjury (Figure 3B), with peak proliferation at day 7 (representative images shown in Figure 3C). We previously reported that β_1_-deficient AT2 cells exhibited increased NF-κB inflammatory signaling in the absence of injury (9). As NF-κB activation promotes cell survival and mitosis (23), we next tested whether the observed excess AT2 proliferation was due in part to upregulated NF-κB activation in β_1_-deficient AT2 cells. We measured AT2 proliferation in LPS-treated precision-cut lung slices (PCLS) generated from β_1_^AT2-KO^ and β_1_^fl/fl^ lungs in the presence/absence of the NF-κB inhibitor BAY 11-7082. Proliferation was detected after 48 hours of treatment using incorporated BrdU, colocalized with pro–SP-C in immunostained PCLS. We verified induction of NF-κB activation with LPS ex vivo in PCLS by immunostaining for nuclear phosphorylated p65, signaling mediator in NF-κB activation (Supplemental Figure 5A). Consistent with in vivo LPS dosing, β_1_^AT2-KO^ PCLS exhibited increased AT2 proliferation with LPS treatment (Figure 4, A and B, individual values presented in Supplemental Figure 5B), which was mitigated by concurrent NF-κB inhibition. For β_1_^fl/fl^ PCLS, AT2 proliferation rates remained similar in all 4 conditions. These data suggest that increased AT2 proliferation in β_1_^AT2-KO^ lungs postinjury is in part due to excess NF-κB activation intrinsic to β_1_-null AT2 cells.

Analyzing cell survival by TUNEL assay co-immunostained with pro–SP-C, we identified a small but significant increase in the total number of apoptotic AT2 cells per field in β_1_^AT2-KO^ lungs on day 21 after LPS (Supplemental Figure 6, A–C). Although we were not able to capture large-scale AT2 apoptosis by TUNEL assay at these time points, early AT2 apoptosis remains a possibility. Further, the near absence of AT1 cells 7 days after injury strongly argues that AT1 cell death occurred early during acute LPS-induced injury.

### Overabundant AT2 cells are transcriptionally distinct during repair in β_1_^AT2-KO^ mice.

**Figure 5:**
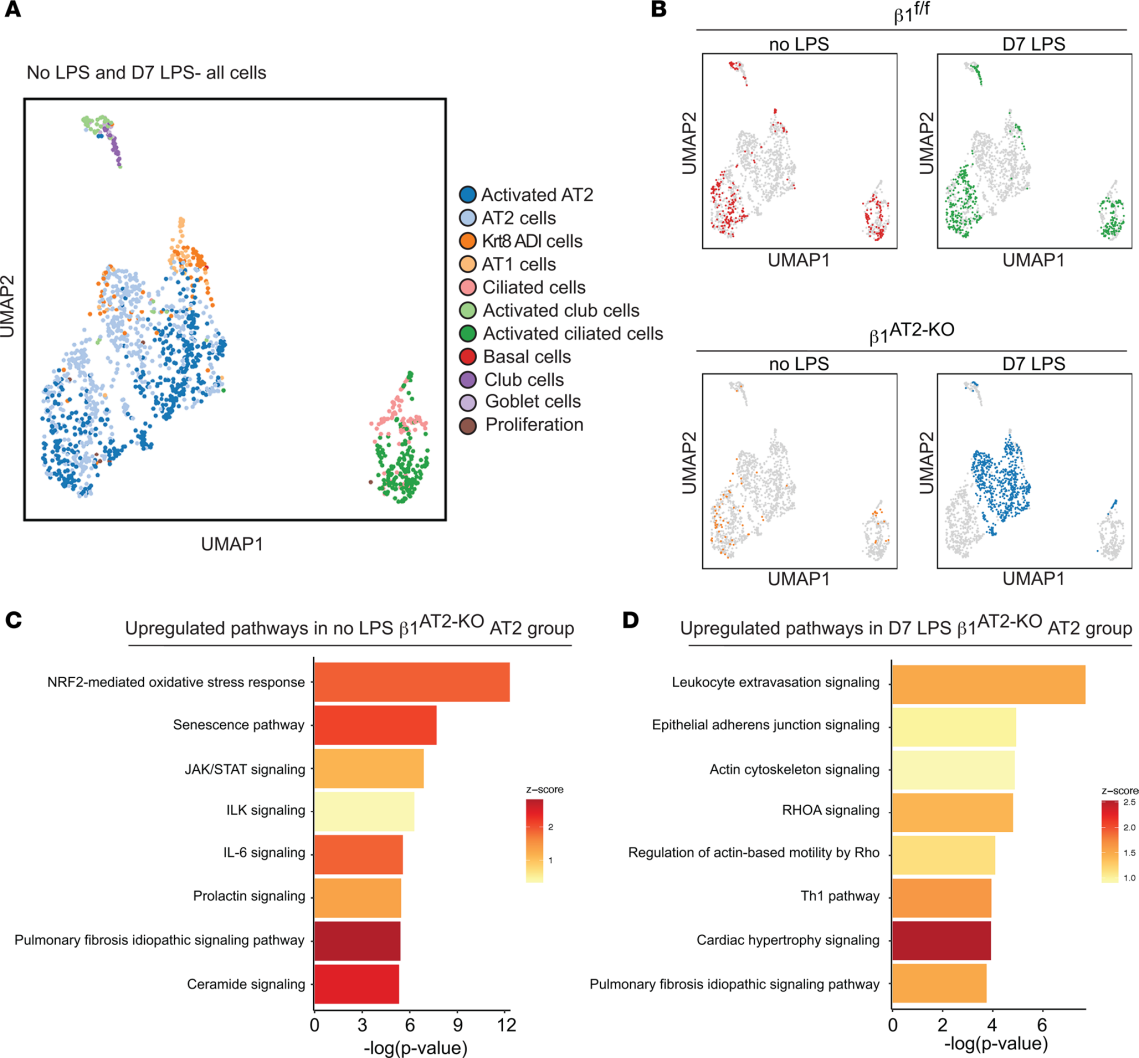
Overabundant AT2 cells are transcriptionally distinct during repair in β_1_^AT2-KO^ mice. (**A**) Uniform manifold approximation and projection (UMAP) of all epithelial cells from β_1_^fl/fl^ and β_1_^AT2-KO^ lungs with/without LPS clustered by label transfer from Strunz et al. (11). (**B**) Individual epithelial populations by group reveal transcriptionally distinct AT2s and activated AT2s in day 7 LPS-treated β_1_^AT2-KO^ lungs. (**C**) Ingenuity Pathway Analysis (QIAGEN) on combined AT2 groups from uninjured β_1_^fl/fl^ and β_1_^AT2-KO^ lungs demonstrates upregulation of oxidative stress, senescence, and inflammatory pathways in β_1_^AT2-KO^ lungs compared with β_1_^fl/fl^ lungs. (**D**) Ingenuity Pathway Analysis shows upregulation of actin cytoskeleton signaling pathways in β_1_^AT2-KO^ AT2 cells compared with β_1_^fl/fl^ AT2 cells at 7 days after LPS treatment.

To investigate the transcriptional phenotype of β_1_-deficient epithelial cells that could explain the development of emphysema and other features of abnormal repair in β_1_^AT2-KO^ mice, we performed single-cell RNA sequencing (scRNA-Seq) on unchallenged and LPS-treated β_1_^fl/fl^ and β_1_^AT2-KO^ lungs, 7 days after LPS exposure. From single-cell suspensions from digested whole lung (*n* = 3–4 mice/group pooled into a single sample for each condition), we collected CD45^–^Ter119^–^ viable cells by fluorescence-activated cell sorting (FACS), thereby excluding immune and red blood cells. We performed scRNA-Seq using the 10x Genomics platform, and doublet removal, normalization, and scaling led to 2,926 cells being analyzed. Initially, we integrated our data set with a recently published scRNA-Seq data from LPS injury (12), and we found similar alveolar epithelial cell types in both data sets (Supplemental Figure 7). To further define our epithelial populations throughout alveolar repair, we analyzed our transcripts utilizing the epithelial markers reported by Strunz et al. with a label transfer technique (11). In both LPS-treated and uninjured mice, we identified all major alveolar epithelial populations in both β_1_^AT2-KO^ and β_1_^fl/fl^ lungs (Figure 5A). Despite differential AT1 cell number difference in injured β_1_^AT2-KO^ lungs by histology, AT1 cells were isolated in low abundance in all 4 groups, uninjured and LPS-treated β_1_^AT2-KO^ and β_1_^fl/fl^ lungs, likely due to cell loss by FACS because of their relatively fragile structure (24). UMAP embedding identified a distinct epithelial population in β_1_^AT2-KO^ lungs 7 days after injury (Figure 5B). Visualizing the UMAPs of the 4 individual groups, most of the epithelial cells were classified as AT2 cells or activated AT2 cells, a cell state marked by inflammation-induced genes. In LPS-treated β_1_^AT2-KO^ lungs, the mixed AT2 and activated AT2 cells were transcriptionally distinct from the other 3 groups. Because this defined β_1_^AT2-KO^ cluster comprised both AT2 and activated AT2 cells, we combined these populations for further analysis in all 4 groups. We examined the top differentially expressed genes in the combined AT2 groups between uninjured and LPS-treated β_1_^AT2-KO^ or β_1_^fl/fl^ mice (Figure 5, C and D, and Supplemental Figure 8, A and B). In uninjured mice, the most enriched pathways were an upregulation of the oxidative stress response pathway in the β_1_^AT2-KO^ AT2 group, followed by senescence, JAK/STAT signaling, and IL-6 signaling pathways. All 4 of these top pathways are relevant to emphysema and lung senescence (25, 26), suggesting baseline conditions that favor accelerated aging in these 3-month-old mice. In LPS-injured lungs from both genotypes, the top 8 differentially upregulated pathways included regulatory signaling networks related to adherens junctions, actin cytoskeleton, and Rho GTPases. As remodeling of adherens junctions is necessary for elongation of cell shape and Rho GTPases are intermediary signaling effectors between integrins and the actin cytoskeleton, these data suggest that β_1_ integrin regulates the transition in cell shape during AT1 cell differentiation.

### During alveolar repair, β_1_ integrin regulates actin expression and RhoA GTPase activation.

**Figure 6:**
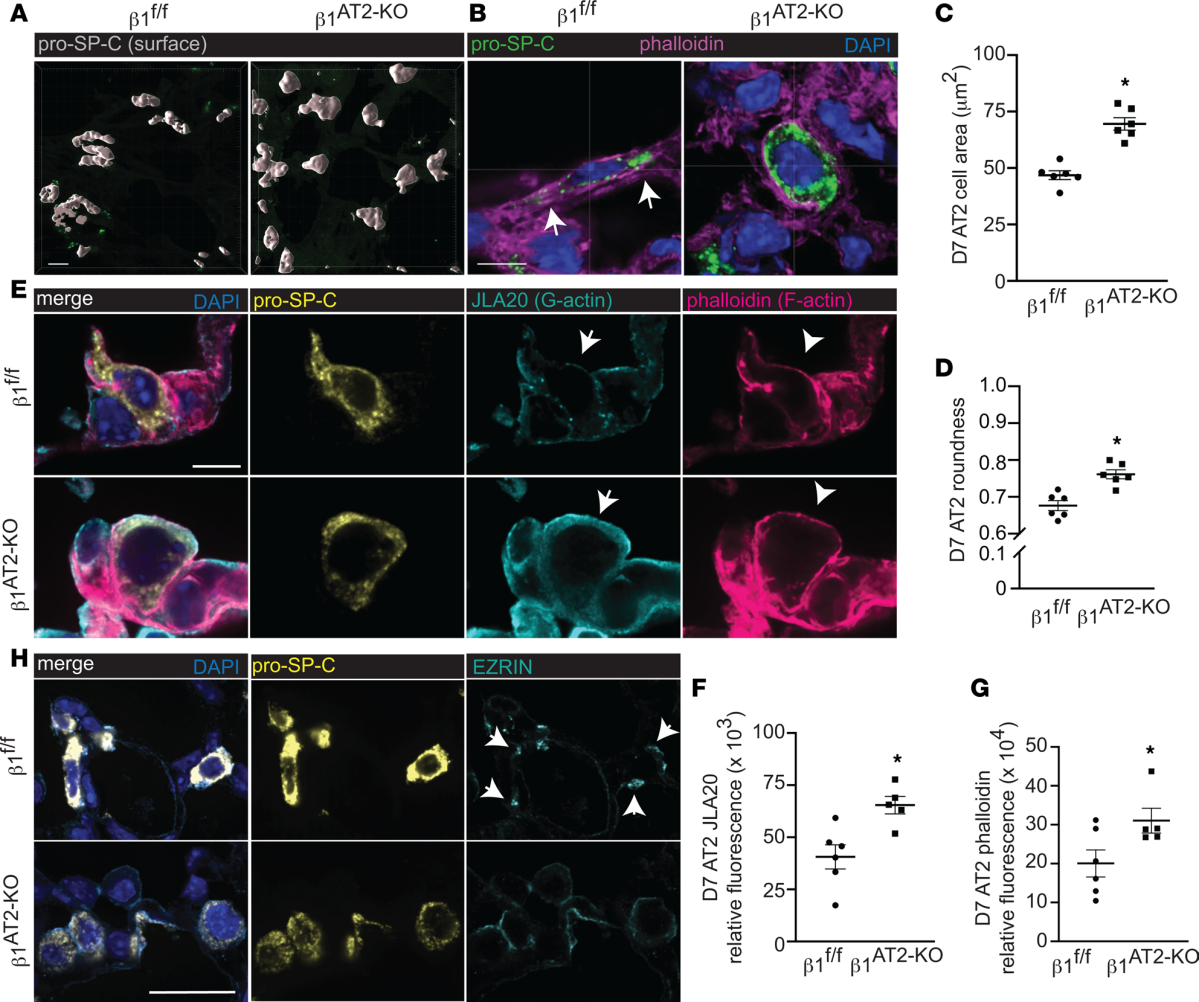
During alveolar repair, β_1_ integrin regulates actin localization and RhoA GTPase activation. (**A**) Surface rendering high-power images of pro–SP-C–immunostained, thick, frozen sections from day 7 (D7) LPS-treated β_1_^fl/fl^ and β_1_^AT2-KO^ lungs. (**B**) High-power images of thick, frozen sections from D7 LPS-treated β_1_^fl/fl^ and β_1_^AT2-KO^ lungs immunostained for pro–SP-C (green) with phalloidin F-actin probe (magenta); arrows indicate areas of actin-rich lateral protrusions. (**C**) Area of pro–SP-C^+^CD68^–^ AT2 cells from D7 LPS-treated β_1_^fl/fl^ and β_1_^AT2-KO^ mice (46.8 ± 2.0 μm^2^ in β_1_^fl/fl^ lungs compared with 69.6 ± 2.8 μm^2^ in β_1_^AT2-KO^ lungs, *n* = 6 mice/group, ≥40 cells measured/mouse imaged from 5 sections, 2-tailed *t* test, *P* < 0.0001). (**D**) Roundness score calculated from pro–SP-C^+^CD68^–^ cells from D7 LPS-treated β_1_^fl/fl^ and β_1_^AT2-KO^ mice (38–60 cells measured/mouse from 5 sections, *n* = 6 mice/group, 2-tailed *t* test comparing genotypes, *P* = 0.0009). (**E**) High-power images of frozen sections prepared at D7 after LPS β_1_^fl/fl^ and β_1_^AT2-KO^ lungs immunostained for pro–SP-C (gold) with JLA20 (cyan) and phalloidin (magenta) probes applied to detect G-actin and F-actin, respectively. Membrane localization of G-actin denoted by arrows and F-actin by arrowheads. (**F** and **G**) Quantification of JLA20 (F) and phalloidin (G) expression in pro–SP-C^+^ AT2 cells in β_1_^fl/fl^ and β_1_^AT2-KO^ lungs D7 after LPS (*n* = 5–6 mice/group, 10 sections/mouse, 2-tailed *t* test with *P* = 0.0088 for JLA20 and *P* = 0.0482 for phalloidin). (H) Representative high-power images from D7 LPS-treated β_1_^fl/fl^ and β_1_^AT2-KO^ lungs immunostained for ezrin (cyan) with AT2 cells identified by RNA in situ hybridization for *Sftpc* (gold). Arrows indicate ezrin expression localized to lateral extensions in β_1_^fl/fl^ AT2 cells, whereas diffuse, nonfocal ezrin expression along the cell membrane is seen in β_1_^AT2-KO^ AT2 cells. * *P* < 0.05. Scale bar = 5 μm for **A**, **B**, and **E**; scale bar = 25 μm for panels in **H**.

**Figure 7:**
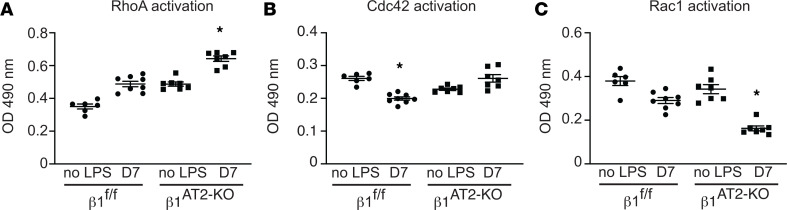
During repair, β_1_ integrin regulates GTPase activation in AT2 cells. (**A**–**C**) GTPase activation assay performed on AT2 cell lysates collected from uninjured and D7 LPS-treated β_1_^fl/fl^ and β_1_^AT2-KO^ lungs (*n* = 6–8 mice/group for each assay; in **A** RhoA 1-way ANOVA * *P* < 0.0001, *F* value 53.42, *df* = 3; in **B** Cdc42 1-way ANOVA * *P* < 0.001, *F* value 17.46, *df* = 3; in **C** Rac1 1-way ANOVA * *P* < 0.0001, *F* value 31.09, *df* = 3).

To investigate how β_1_ integrin regulates the actin cytoskeleton during alveolar repair, we examined AT2 cell shape by confocal microscopy. Surface rendering of pro–SP-C^+^ AT2 cells demonstrated a field of globular AT2 cells in β_1_^AT2-KO^ lungs, whereas β_1_^fl/fl^ pro–SP-C^+^ AT2 cells exhibited a flattened, elongated cell shape (Figure 6A). We next imaged 50 μm cryosections detecting F-actin with phalloidin probe concurrent with pro–SP-C immunodetection. The injured β_1_^fl/fl^ pro–SP-C^+^ AT2 cells formed actin-based protrusions at the areas of their lateral extensions (arrows in Figure 6B), a feature shared with differentiating AT2 (to AT1) epithelial progenitors during development (27). By contrast, AT2 cells in injured β_1_^AT2-KO^ lungs were larger and rounded with cortical F-actin. We quantified AT2 cell shape at 7 days postinjury by calculating cell area and a cell roundness score. Given the persistent macrophage inflammation during repair in LPS-treated β_1_^AT2-KO^ lungs, we specifically excluded the occasional macrophage that has efferocytosed an injured pro–SP-C^+^ cell from this analysis by calculating the day 7 cell shape metrics on pro–SP-C^+^CD68^–^ cells only (Supplemental Figure 9A). AT2 cells were significantly larger in injured β_1_^AT2-KO^ lungs (66.8 ± 3.0 μm^2^) compared with β_1_^fl/fl^ lungs (48.4 ± 1.8 μm^2^) (Figure 6C). Since lateral extension is necessary for cells to transition from AT2 to AT1 and we demonstrated that cell shape is β_1_ dependent in AT2 cells, we calculated a roundness score, which quantifies the degree of smooth cellular contours in an unbiased manner (28). The roundness score in β_1_^AT2-KO^ AT2 cells on day 7 after injury, as they failed to elongate and change shape into flattened AT1 cells, was significantly increased compared with β_1_^fl/fl^ AT2 cells (Figure 6D). AT2 cells from LPS-treated β_1_^AT2-KO^ lungs maintained an elevated roundness score throughout repair, based on pro–SP-C^+^ cells (Supplemental Figure 9B). This finding is consistent with an inability to form lateral extensions and impaired actin remodeling. By contrast, β_1_^fl/fl^ AT2 cell roundness decreased at times of rapid differentiation during repair, indicative of differentiation into a flattened AT1 cell shape. Since our scRNA-Seq data suggested upregulation of actin/cytoskeleton signaling pathways, we next applied a G-actin probe, JLA20, along with F-actin phalloidin and pro–SP-C to study actin remodeling (Figure 6E). We found increased G-actin and F-actin in injured β_1_^AT2-KO^ lungs, with F-actin primarily cortically localized in the β_1_^AT2-KO^ lungs. We verified these increases by calculating the relative fluorescence of the JLA20 and phalloidin probes in pro–SP-C^+^ cells using corrected total cell fluorescence to normalize fluorescence to total cell area (Figure 6, F and G). Because actin remodeling and localization are mediated in part by the cytoskeletal protein ezrin, we immunodetected ezrin in β_1_^AT2-KO^ and β_1_^fl/fl^ lungs on day 7 postinjury combined with *Sftpc* RNA in situ hybridization to mark AT2 cells. Ezrin was as expected localized to points of lateral cellular protrusion in elongating AT2 cells in control mice (Figure 6H) while being much more widespread along the cell membrane in AT2 cells in β_1_^AT2-KO^ lungs, suggesting an impaired ability to direct actin localization in concert with loss of lateral elongation. We validated the linkage to small GTPases (29) suggested by scRNA-Seq analysis above using protein assays for specific GTPases. AT2 cells from LPS-injured β_1_^AT2-KO^ lungs exhibited increased RhoA and Cdc42 GTPase activation, as would be anticipated for a cell undergoing efficient actin remodeling (Figure 7, A and B). By contrast, GTPase activation was decreased for Rac1 (Figure 7C), a related GTPase involved in forming smaller actin-based structures such as microvilli. Taken together, these data indicate RhoA and Cdc42 GTPase activation in AT2 cells is enhanced in β_1_ integrin–deficient AT2 cells, as would be expected for a cell with active actin remodeling. However, evidence of actin remodeling at the site of lateral extension is absent, suggesting a block downstream of GTPase signaling events propagated by the β_1_ intracellular tail in β_1_^AT2-KO^ lungs. Thus, β_1_ integrin is a critical component for proper actin production and localization in differentiating AT2 cells during repair.

### Postinjury β_1_-deficient AT2 cells exhibit an AT2-AT1 mixed transcriptomic phenotype.

**Figure 8:**
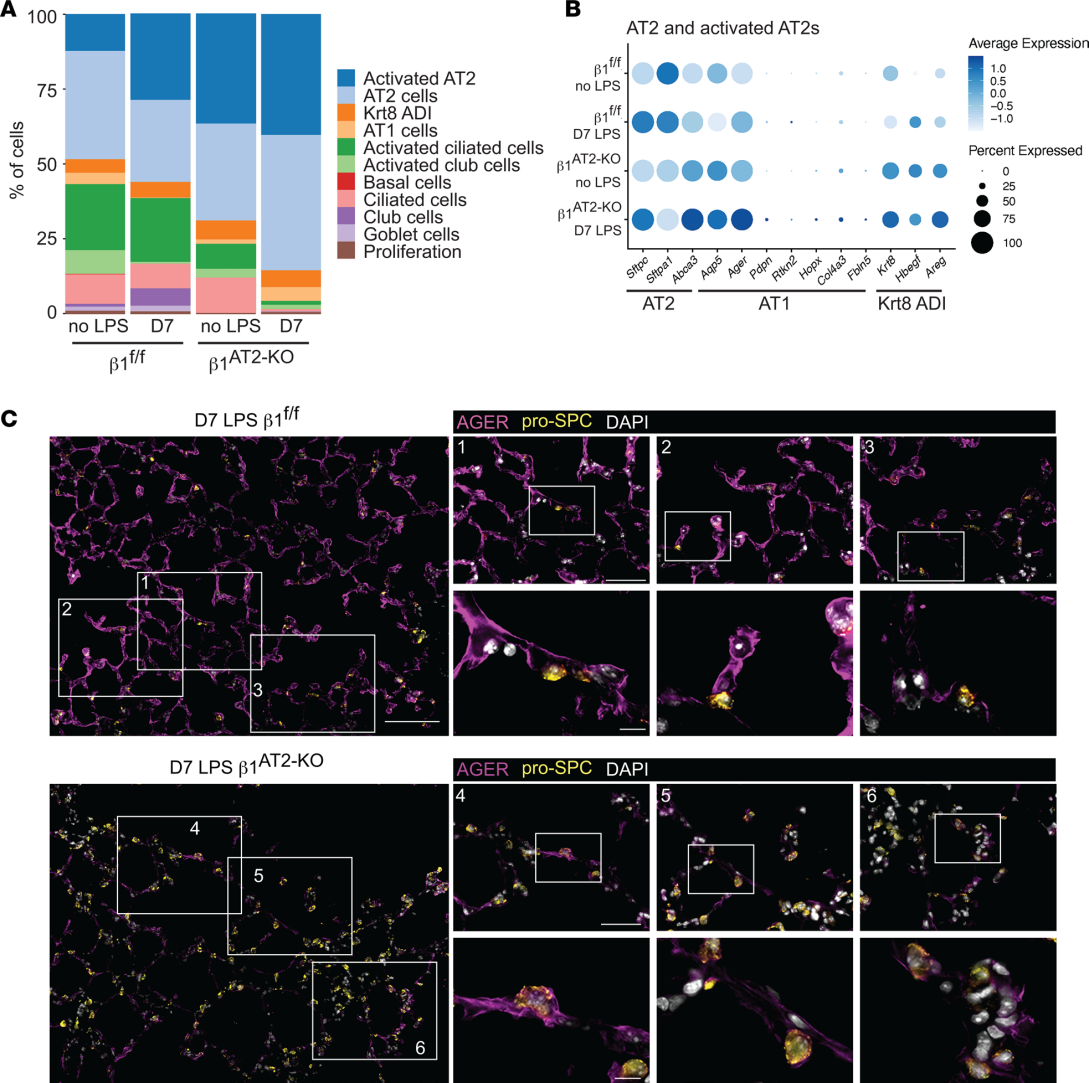
Postinjury β_1_-deficient AT2s exhibit an AT2-AT1 mixed epithelial transcriptomic phenotype. (**A**) Stacked bar graph of epithelial proportions demonstrates an expansion of the AT2 and activated AT2 populations in day 7 (D7) LPS-treated β_1_^AT2-KO^ lungs. (**B**) Marker gene expression by genotype and treatment group in AT2/activated AT2 cluster, in which higher expression is represented with a darker color and the size of the dot reflects the proportion of cells expressing that marker. (**C**) Representative low-power images from day 7 LPS-treated lung sections immunostained for the AT1 marker AGER (purple) and AT2 marker pro–SP-C (gold) demonstrate overall decreased AGER in β_1_^AT2-KO^ lungs. Three insets per low-power field show colocalization of AGER with pro–SP-C^+^ AT2 cells in D7 β_1_^AT2-KO^ lungs. Scale bar = 100 μm for low-power image in **C**, 50 μm for middle inset, and 10 μm for high-power inset.

Our observation of increased AT2 cells and decreased AT1 cells suggested impairment in AT2-to-AT1 differentiation during alveolar repair. To define this defect at the transcriptional level, we quantified lung epithelial cell subtypes as previously defined by Strunz et al. (11). Consistent with our histology of the LPS-injured mice, there was a significant increase in the representation of AT2 and activated AT2 cells in day 7 post–LPS injury β_1_^AT2-KO^ lungs but not control β_1_^fl/fl^ (Figure 8A). We next compared the AT2, AT1, and cytokeratin 8^+^ “intermediate cell” transcriptomic profiles in our uninjured and LPS-treated β_1_^AT2-KO^ and β_1_^fl/fl^ lungs. Strunz et al. (11) defined an intermediate cell state with features of both AT2 and AT1 cells as a “Krt8-positive alveolar differentiation intermediate (ADI).” Both uninjured and LPS-treated β_1_^AT2-KO^ and β_1_^fl/fl^ AT2/activated-AT2 cells expressed AT2 hallmark genes (e.g., *Sftpc, Sftpa1,* and *Abca3*), as expected (Figure 8B). The Krt8^+^ ADI hallmark genes *Krt8, Hbegf,* and *Areg* were moderately increased in uninjured β_1_^AT2-KO^ AT2 cells, and this increase was accentuated after LPS. By contrast, there was minimal expression of these markers in uninjured or LPS-treated β_1_^fl/fl^ AT2 cells. Finally, uninjured β_1_^AT2-KO^ AT2/activated AT2 cells exhibited enhanced AT1 cell marker expression compared with uninjured β_1_^fl/fl^ AT2 cells, which was also accentuated post-LPS. We validated increased AT1 marker expression in injured β_1_-deficient pro–SP-C^+^ AT2 cells by co-immunostaining for advanced glycosylation end product-specific receptor (AGER), the AT1 marker with the highest expression by our transcriptional analysis. In injured β_1_^fl/fl^ lungs, AGER localized to the typical AT1 distribution throughout the lung parenchyma, largely distinct from pro–SP-C^+^ AT2 cells (Figure 8C, insets 1–3). By contrast, injured β_1_^AT2-KO^ lungs exhibited notably less AGER throughout the lung parenchyma compared with β_1_^fl/fl^ lungs. Importantly, the small amount of AGER present in β_1_^AT2-KO^ lungs colocalized with large, rounded pro–SP-C^+^ AT2 cells (Figure 8C, insets 4–6, single-channel images in Supplemental Figure 10). The robust acquisition of AT1 markers in injured, rounded, large β_1_^AT2-KO^ AT2 cells suggests that requisite β_1_ integrin–dependent mechanisms, such as cell shape change, occur subsequent to initial AT1 marker acquisition. In summary, the absence of β_1_ increases the proportional representation of intermediate cell state markers, even in cells that remain classified as AT2 cells, indicating a significant block in the progression of differentiation.

### Mixed-phenotype AT2 cells persist, proliferate, and maintain an enlarged, rounded cell shape in late alveolar repair.

**Figure 9:**
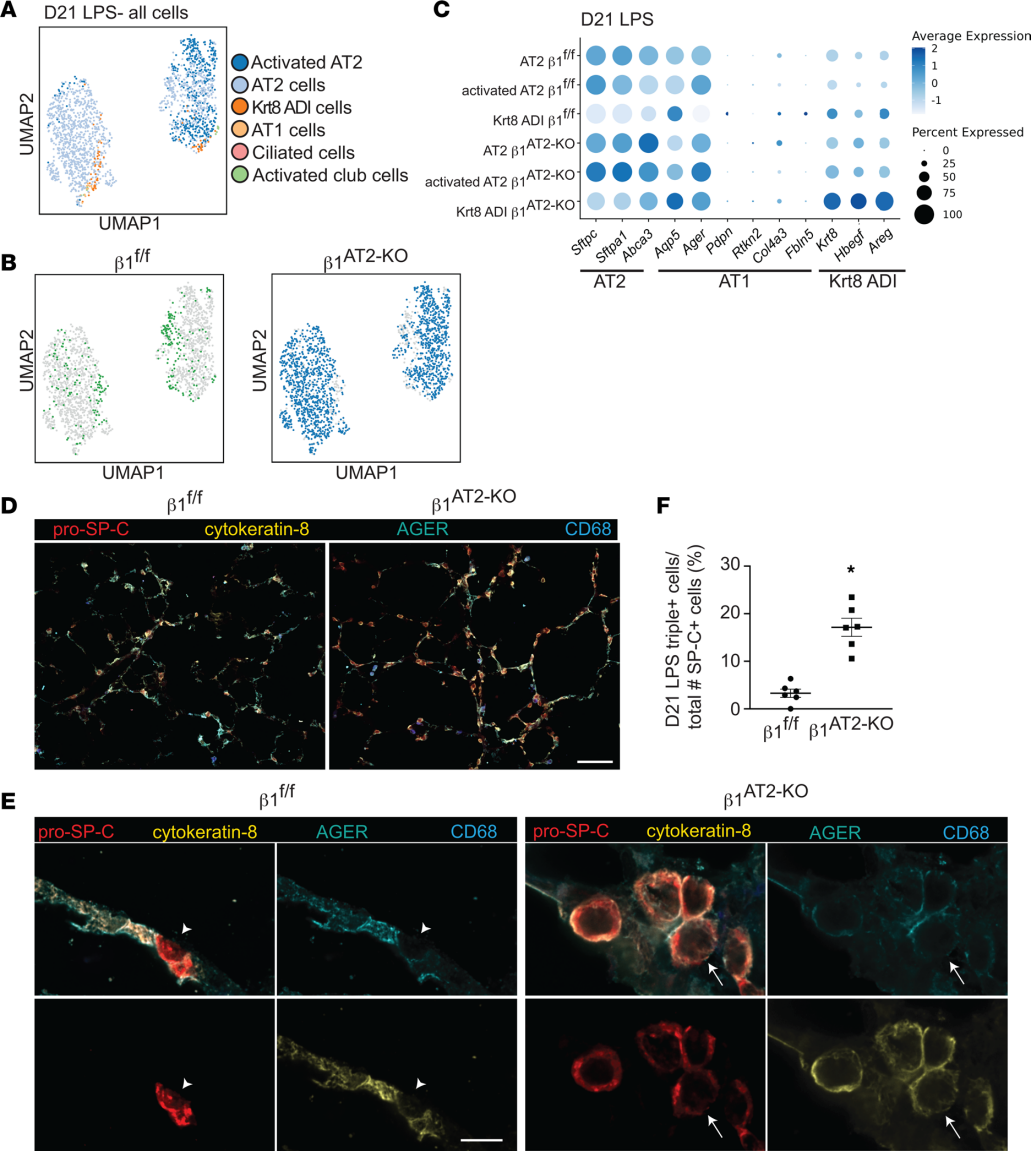
AT2s of mixed transcriptomic phenotype persist, proliferate, and maintain an enlarged, rounded cell shape in late alveolar repair. (**A**) UMAP of all epithelial cells from β_1_^fl/fl^ and β_1_^AT2-KO^ lungs 21 days after LPS treatment clustered by label transfer from Strunz et al. (11). (**B**) Individual epithelial populations by group reveal transcriptionally abundant AT2s and activated AT2s in day 21 LPS-treated β_1_^AT2-KO^ lungs. (**C**) Hallmark gene expression by genotype in AT2, activated AT2, and Krt8 ADI clusters, in which higher expression is represented with a darker color and the size of the dot reflects the proportion of cells expressing that marker. (**D**) Representative low-power images of β_1_^fl/fl^ and β_1_^AT2-KO^ lungs 21 days after LPS co-immunostained for pro–SP-C (red), cytokeratin 8 (gold), AGER (cyan), and CD68 (blue). β_1_^AT2-KO^ lungs are notable for enlarged airspaces and increased numbers of round, large pro–SP-C^+^ AT2 cells, which are distinct from alveolar macrophages. (**E**) High-power images of lung sections immunostained for pro–SP-C, cytokeratin 8, AGER, and CD68, as above, demonstrate round, large pro–SP-C^+^ AT2 cells that colocalize with cytokeratin 8, with a subset also triple positive (pro–SP-C^+^cytokeratin 8^+^AGER^+^). Arrow denotes occasional triple-positive cells in β_1_^AT2-KO^ lungs, and arrowhead marks AGER^–^ AT2 cells in β_1_^fl/fl^ lungs. (F) Quantification of pro–SP-C^+^cytokeratin 8^+^AGER^+^ triple-positive cells as a percentage of total pro–SP-C^+^ cells in day 21 LPS-treated lungs (*n* = 6 mice/group, 10 original magnification, 60×, sections/mouse, *P* < 0.0001 by 2-tailed *t* test). * *P* < 0.05. Scale bar = 100 μm in **D**, 10 μm in **E**.

Since our histological and scRNA-Seq analysis showed impaired AT2 differentiation at day 7 post-LPS, we characterized the blockage in differentiation in late repair. As described above, we performed scRNA-Seq on uninjured and LPS-treated β_1_^fl/fl^ and β_1_^AT2-KO^ lungs, 21 days post-LPS. Knowing that alveolar epithelial cells of mixed transcriptomic phenotype exist at day 7 post-LPS in β_1_^AT2-KO^ lungs, we investigated persistence of these cells at day 21 post-LPS by RNA-Seq of CD45^–^Ter119^–^ single-cell suspensions enriched for CD326^+^ epithelial cells. UMAP embedding with label transfer from Strunz et al. (11) revealed similarities between β_1_^fl/fl^ and β_1_^AT2-KO^ alveolar cells (Figure 9, A and B). By pro–SP-C/Ki-67 co-immunostaining, we demonstrated increased AT2 proliferation even during late repair. Our scRNA-Seq data verified that the AT2 population from day 21 LPS-treated β_1_^AT2-KO^ lungs exhibited a significantly increased G2M proliferation score compared with AT2 cells from day 21 LPS-treated β_1_^fl/fl^ lungs (Supplemental Figure 11A). We next analyzed the expression of AT2, Krt8^+^ ADI, and AT1 hallmark genes in day 21 LPS-treated β_1_^AT2-KO^ and β_1_^fl/fl^ alveolar epithelial cells. We identified enhanced AT2 markers, *Sftpc*, *Sftpa1,* and *Abca3*, in AT2 and activated AT2 cell populations from β_1_^AT2-KO^ mice compared with β_1_^fl/fl^ mice (Figure 9C). In addition, β_1_^AT2-KO^ Krt8^+^ ADI cells retained AT2 hallmark gene expression compared with β_1_^fl/fl^ Krt8^+^ ADI cells, exhibited markedly enhanced expression of the 3 intermediate state markers (*Krt8, Hbegf,* and *Areg*), and showed increased expression of AT1 hallmark genes, *Aqp5* and *Ager*. These β_1_^AT2-KO^ Krt8^+^ ADI cells simultaneously expressed transcriptional markers from all 3 stages of transition, providing a transcriptional rationale for failed repair in β_1_-deficient mice. We validated the persistent mixed transcriptional phenotype in β_1_^AT2-KO^ lungs by immunodetection. In day 21 LPS-treated β_1_^AT2-KO^ lungs, remodeled areas exhibited numerous large, rounded pro–SP-C^+^cytokeratin 8^+^ cells with less AGER in the AT1 distribution compared with injured β_1_^fl/fl^ lungs (Figure 9D). High-power images demonstrated pro–SP-C^+^cytokeratin 8^+^ cells with occasional cells also positive for the AT1 marker AGER (arrows in Figure 9E). By contrast, triple-positive cells (pro–SP-C^+^cytokeratin 8^+^AGER^+^) were rare in injured β_1_^fl/fl^ lungs. The number of triple-positive cells was quantified as a percentage of total pro–SP-C^+^ cells (Figure 9F). Given the disparate cellular morphology of β_1_-deficient AT2 cells at earlier time points, we quantified differences in cell area in pro–SP-C^+^ AT2 cells. At 21 days after LPS, injured AT2 cells from β_1_^AT2-KO^ lungs remained enlarged with a significantly increased cell area (Supplemental Figure 11B). These findings demonstrate that β_1_-mediated ECM interactions are essential during alveolar repair for the maintenance of alveolar structure, epithelial cell shape change, and progression of their transcriptomic phenotype.

### AT2 cells deficient in β_1_ fail to repopulate the alveolus with AT1 morphology.

**Figure 10:**
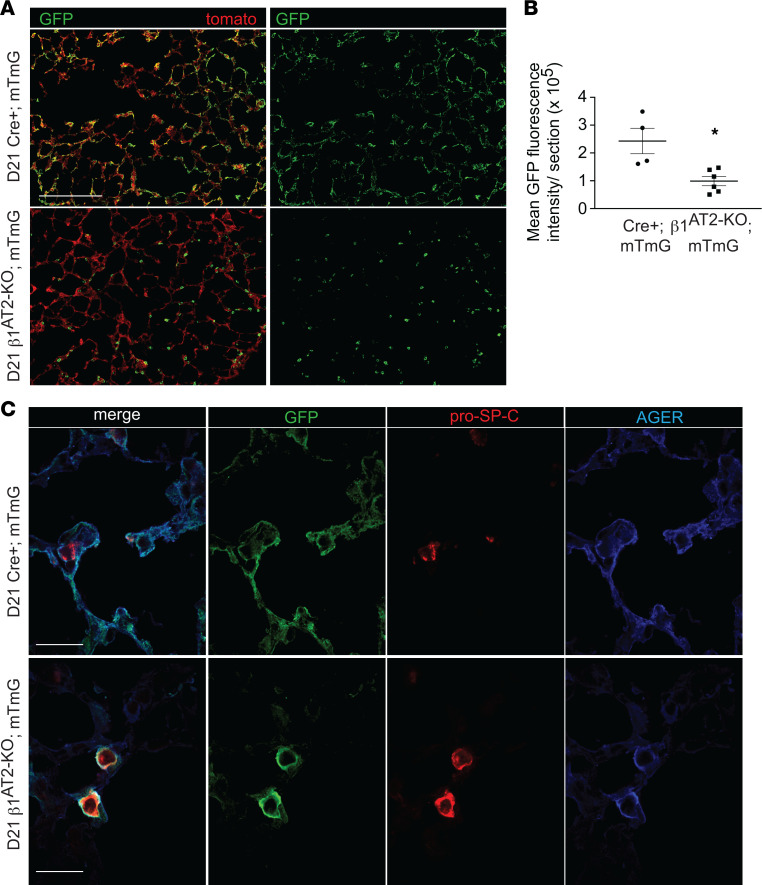
β_1_^AT2-KO^ mice fail to repopulate alveolus with cells of AT1 morphology. (**A**) Low-power images of Cre^+^ mTmG control mice demonstrate Cre-recombinase reporter–labeled GFP^+^ cells of both AT1 and AT2 cell shape repopulate the injured alveolus 21 days after LPS. GFP-labeled cells in β_1_^AT2-KO^ mTmG mice retain only AT2 morphology and fail to attain an AT1 cell shape at 21 days. (**B**) Quantification of mean GFP fluorescence intensity shows decreased area of lung repopulated by GFP-labeled cells at 21 days post-LPS in β_1_^AT2-KO^ mTmG mice (*n* = 4 Cre^+^ mTmG and 6 β_1_^AT2-KO^ mTmG mice; 10 original magnification, 20×, sections/mouse, *P* = 0.0087 by 2-tailed *t* test). (C) High-power images of Cre^+^ mTmG and β_1_^AT2-KO^ mTmG lung sections immunostained for pro–SP-C (red) and AGER (blue); tomato omitted in imaging. Large, round GFP^+^-labeled cells acquire both AT2 (pro–SP-C^+^) and AT1 (AGER^+^) markers in β_1_^AT2-KO^ mTmG lungs. GFP^+^-labeled cells possess either AT2 or AT1 markers at 21 days post-LPS in Cre^+^ mTmG lungs. * *P* < 0.05. Scale bar = 200 μm in **A** and 25 μm in **C**.

To prove that AT2 cells from β_1_^AT2-KO^ mice cannot repopulate the alveolus after injury, we crossed β_1_^AT2-KO^ mice with the mTmG Cre-recombinase reporter, labeling Cre-recombinase active AT2 cells beginning at P28. Mice were then maintained on doxycycline for a month and treated with LPS at 3 months of age. We verified the presence of GFP-labeled AT2 cells prior to injury in β_1_^AT2-KO^ mTmG and Cre^+^ mTmG mice (Supplemental Figure 12A). As anticipated, histological examination of lungs harvested 21 days after LPS demonstrated GFP^+^ cells in both AT2 and AT1 distributions in all the control Cre^+^ mTmG mice (Figure 10A), indicating patches of AT2-to-AT1 alveolar repopulation postinjury. By contrast, GFP-labeled cells in β_1_^AT2-KO^ mTmG lungs retained almost exclusively a rounded AT2 morphology, with only rare acquisition of an elongated AT1 cell shape. We quantified the degree to which GFP-labeled AT2 cells repopulated injured β_1_^AT2-KO^ mTmG and Cre^+^ mTmG lungs by fluorescence intensity per low-power field section (Figure 10B and Supplemental Figure 12B). Immunostained sections for pro–SP-C and AGER verified the rounded GFP^+^ cells in β_1_^AT2-KO^ mTmG lungs were pro–SP-C^+^, and although rounded GFP^+^ cells were also frequently AGER^+^ (Figure 10C), very few thin GFP^+^AGER^+^pro–SP-C^+^ cells were observed in β_1_^AT2-KO^ mTmG. In Cre^+^ mTmG lungs, GFP labeling colocalized with AGER^+^ thin AT1 cells, verifying successful AT2-to-AT1 differentiation in control mice. These data demonstrate failed AT2-to-AT1 differentiation associated with impaired cell shape change and incomplete phenotypic epithelial marker differentiation in the absence of epithelial β_1_ integrin.

## Discussion

### β_1_ Integrin is exquisitely required for differentiation during repair, in contrast to development or alveolar homeostasis.

Since progenitor AT2 proliferation precedes differentiation in the injury repair cycle, the overabundance of incompletely differentiated AT2 cells in β_1_^AT2-KO^ mice reveals a substantial block in differentiation postinjury. We previously reported increased AT2 proliferation with deletion of epithelial β_1_ in both the developing and adult lung in the absence of injury (8, 9). The degree of AT2 accumulation in β_1_-deficient repair reported here far exceeds the developmental and homeostatic contexts. This relative difference in AT2 surplus, coupled with successful AT2-to-AT1 differentiation in uninjured, aged β_1_^AT2-KO^ mice (9), shows that β_1_-ECM interactions are even more critical for differentiation during repair of adult injury than during development or homeostasis. This comparison suggests that β_1_-dependent differentiation mechanisms are specifically adjusted to fit the requirements of the peripheral stem cell niche. One possible explanation is that the time interval over which cells change shape and differentiate differs between the 3 contexts of development, homeostasis, and repair. Developmental AT2-to-AT1 differentiation occurs over weeks, and sporadic AT2 replacement of AT1 cells in the adult lung occurs without the urgency of replacing a widely denuded BM. By contrast, AT2 differentiation post-LPS happens en masse with the BM exposed within hours and repopulated within days. This shorter interval makes robust upregulation of facultative differentiation pathways less likely, exposing a dependence on β_1_-mediated mechanisms for regulating differentiation. Another possible reason for the failed repair in β_1_^AT2-KO^ mice after LPS is the presence of preexisting homeostatic defects. Uninjured β_1_^AT2-KO^ mice exhibit impaired epithelial tight junctions and chronic inflammation (9). These conditions exacerbate injury, contribute to epithelial loss, and heighten demand on repair mechanisms, further exposing the dependence on β_1_-mediated differentiation mechanisms. A third possible reason for the β_1_ requirement during repair may be related to the condition of the BM. It is well known that changes in the relative amounts of ECM components will push the alveolar epithelium toward proliferation or differentiation (30), though less is known about how structural changes to the BM itself drive epithelial behavior in the alveolar stem cell niche. The mature uninjured lung has a relatively stable BM with slow turnover of BM proteins (31). However, during LPS-induced injury, inflammatory cell–associated proteases partially degrade the alveolar BM within hours (3, 4, 32). Numerous proteases degrade ECM components preferentially, thereby significantly modifying the carefully balanced alveolar BM composition (4). As different injuries induce varying types of protease release, damage to the BM components will change depending on type and severity of insult, further shifting β_1_-ECM interactions in the stem cell niche away from homeostatic conditions. In addition, individual β_1_-containing integrin pairs preferentially bind their preferred ECM ligands, thereby setting up a situation where injury-induced changes to β_1_-ECM interactions can either restrict or augment epithelial differentiation.

The progressive increase in AT2 cell number that escalates late in repair in β_1_^AT2-KO^ lungs indicates that β_1_-ECM interactions may serve as a brake on AT2 progenitor cell proliferation and could be a requisite step for progression from proliferation to differentiation during the alveolar injury repair cycle and that this stalled differentiation stimulates a feed-forward loop that creates a continued abnormal call for progenitor proliferation. The lung is unique among branched organs in that there exists a distal (in fact, intra-alveolar) epithelial progenitor representing an indigenous contributor to tissue regeneration (13–15, 33), whereas no such epithelial progenitor has been reported for the kidney, mammary gland, or pancreas. Activation of the NF-κB pathway is pro-survival and pro-proliferative in many contexts, initially reported in hyperproliferative keratinocytes with constitutive NF-κB activation (23). Although multiple signaling pathways have been proposed as positive and negative regulators of AT2 progenitor proliferation in the adult lung (13–16, 34–37), their interaction with the NF-κB pathway in the injured lung remains largely undefined, though a recent study by Sieber et al. reports NF-κB–driven airway proliferation in human idiopathic pulmonary fibrosis airway epithelial/fibroblast cocultures (38). In other organs, β_1_-containing integrins have been reported to negatively regulate epithelial proliferation, with an EGFR activation–dependent mechanism identified in the intestinal epithelium (39), but these studies do not consider progenitor proliferation in the context of repair. Consideration of our data in light of this published work suggests that β_1_ integrin restricts proliferative potential in progenitor AT2 cells in the injured distal lung.

### Failed differentiation in injured β_1_-deficient mice reveals that cell shape change is intimately tied to progression of differentiation.

We demonstrate that β_1_-ECM interactions are a requisite component for epithelial cell shape change through actin remodeling in the injured alveolus. The connection between integrins and cellular extension comes from studies in Drosophila epithelium and murine podocytes, both in the *uninjured* state (40–44). Our scRNA-Seq analysis supports the concept that cell shape change is linked to progression of differentiation, as actin remodeling pathways were enhanced in LPS-treated β_1_-deficient AT2 cells, but differentiation remained blocked with an overabundance of large, rounded AT2 cells. It is possible that loss of integrin clustering at the cell membrane disrupts the spatial specificity of Rho GTPase signaling (45). Certainly, diffuse ezrin expression associated with persistence of a rounded cell shape and loss of lateral cellular extensions observed in LPS-treated β_1_^AT2-KO^ AT2 cells are consistent with impaired actin nucleation.

Historically, cellular identity has been defined by histological shape and directly observable characteristics of function (e.g., lamellar body synthesis by electron microscopy). The advent of single-cell transcriptomics increases the granularity with which we can group similar cells together. While this advance augments our understanding of cellular transitional states, the question of how to define groups of similar cells and the boundary between different cell identities is perhaps murkier. Previously published injury models report similar temporary and resolvable transitional cell states after bleomycin injury and other inflammatory stimuli (17, 18), which are marked by high *Krt8* expression. Our transcriptomic data demonstrated persistently blocked AT2-to-AT1 differentiation in injured β_1_^AT2-KO^ lungs, suggesting that they are stuck in a transitional state even in late repair. Although we believe our model is unique in the degree and persistence of failed differentiation, our transcriptomic data have some similarities and differences with recently published single-cell sequencing data from LPS injury (12). The observed differences in the transitional cell state populations identified are possibly linked to technical differences in how epithelial cells were sorted; Riemondy et al. used AT2 cells selected by expression of a fluorescent reporter for *Sftpc* expression prior to scRNA-Seq, removing transitional cells with lower *Sftpc* expression.

### Loss of β_1_-mediated epithelium-ECM interactions increases susceptibility for emphysematous abnormal repair and accelerates aging.

Loss of β_1_ integrin in AT2 cells generates a senescent transcriptional signature in an otherwise uninjured youthful lung that translates into emphysematous abnormal repair with injury. Emphysema is a principal component of chronic obstructive pulmonary disease (COPD), one of the most common human pulmonary diseases that increases in incidence with age (46, 47). Although airspace expansion has been reported with either two-hit injury models or chronic repetitive low-dose LPS exposure (48–52), our β_1_-deficient mice are the first targeted epithelial deletion to our knowledge that results in subacute alveolar destruction following a single LPS exposure, underscoring the importance of epithelial β_1_ integrin in senescence and its associated pulmonary pathologies. Our scRNA-Seq pathway analysis demonstrates uninjured β_1_-deficient AT2 cells possess an inherent susceptibility to injury, as uninjured β_1_^AT2-KO^ mice exhibit upregulation of oxidative stress, senescence, and inflammatory pathways, all contributing factors to emphysema pathogenesis. LPS-challenged β_1_^AT2-KO^ mice also exhibit increased inflammation during repair, an associated finding in COPD (53), with a sustained increase in neutrophils at day 7 after LPS, potentially making prolonged exposure to elastase a contributing factor for emphysema development. At 21 days postinjury, β_1_^AT2-KO^ lungs retain elevated numbers of macrophages, a significant source of matrix metalloproteinase-9, which remodels alveolar matrix in COPD (50). It is unlikely that increased AT2 apoptosis in β_1_^AT2-KO^ lungs is the primary mechanism for the development of emphysema, as it was only present late in repair at day 21, when emphysematous structural changes had already occurred. The etiology for emphysematous alveolar remodeling in young β_1_-deficient mice is likely multifactorial but rooted in the preexisting upregulation of senescence, oxidative stress, and inflammatory pathways. Additional studies addressing how β_1_ integrin regulates these pathways would elucidate if and how β_1_-dependent mechanisms could increase susceptibility of human COPD.

In conclusion, this study shows that β_1_ integrin–mediated mechanisms are a requisite component of rapid and complete alveolar repair following even mild LPS-induced lung injury and involve regulation of AT2 proliferation, differentiation, and cell shape change. In the latter, we propose that cytoarchitectural changes, such as the formation of actin-rich lateral cellular extensions, are specifically tied to progression of AT2-to-AT1 differentiation during repair. Loss of β_1_ integrin results in upregulation of senescence, oxidative stress, and inflammatory pathways already preinjury, thereby increasing susceptibility to emphysema, a common age-related lung structural deficit.

## Methods

### Mice and LPS injury.

We induced β_1_ integrin in AT2 cells in the adult murine lung by crossing transgenic mice with inducible Cre-recombinase expression by the doxycycline-inducible reverse tetracycline transactivator under control of the SP-C promoter (SP-C rtTA Tet-O-Cre) with integrin β_1_^fl/fl^ mice (54, 55). Littermate rtTA^–^ Tet-O-Cre^–^ negative (β_1_^fl/fl^) mice were used as control mice. Doxycycline drinking water (2 g/L) was administered for 4 weeks beginning at P28 to triple-transgenic SP-C rtTA Tet-O-Cre β_1_^fl/fl^ mice (called β_1_^AT2-KO^ mice) and littermate β_1_^fl/fl^ controls, as previously described (9). Following proposed guidelines to limit Cre leak with the doxycycline-inducible system (20), we used the more faithful line 2 strain, employed a breeding strategy to keep the rtTA allele hemizygous, and utilized an injury model that targets the peripheral lung rather than the airway epithelium. After a 1-month period off doxycycline to minimize any potential toxicity from tissue-stored doxycycline, we challenged 3-month-old β_1_^AT2-KO^ and β_1_^fl/fl^ mice with a single IT dose of LPS or PBS (3 μg/g mouse weight, equivalent volume for PBS) and harvested tissue at the indicated dates. For β_1_^AT2-KO^ and β_1_^fl/fl^ mice administered delayed doxycycline, IT LPS was given at 3 months of age as above, and then doxycycline drinking water was provided from 5 days after LPS until harvest at 21 days after LPS. SP-C rtTA Tet-O-Cre (no floxed alleles) and β_1_^AT2-KO^ mice were crossed to the mTmG Cre-recombinase reporter mice. Doxycycline drinking water and LPS were administered as above. SP-C rtTA, Tet-O-Cre, and mTmG mice were purchased from The Jackson Laboratory. Integrin β_1_^fl/fl^ mice were gifted by Elaine Fuchs (Howard Hughes Medical Institute, The Rockefeller University, New York, New York, USA). All mice were maintained on the C57BL/6 background.

### Histology and morphological analysis.

For histological analysis on paraffin sections, mice were sacrificed, the right ventricle was flushed with PBS, and lungs were inflation fixed at 25 cm with 10% formalin. After paraffin processing, embedding, and sectioning, lungs were hematoxylin and eosin–stained for morphological analysis by mean linear intercept, which was calculated from images (≥10 nonoverlapping images per mouse) obtained using a ×40 objective on a Keyence BZ-X710 inverted fluorescence phase contrast microscope. Immunofluorescence staining was performed on paraffin or frozen sections. Frozen blocks were prepared from lung sections inflation-fixed with a 2:1 PBS/O.C.T. (Tissue-Tek) mixture, embedded, and sectioned at either 8 μm or 50 μm thickness. Frozen section slides were then fixed with 4% paraformaldehyde, permeabilized with 0.1% Triton X-100, and blocked with 5% donkey serum for 2 hours at 37°C. Slides were incubated in primary antibody overnight at 4°C, followed by secondary antibody incubation for 2 hours at room temperature. For sequential primary antibody staining, a second intervening blocking step was utilized. Nuclei were stained with DAPI, ProLong Gold mountant (Thermo Fisher Scientific P36930) was applied, and sections were imaged. High-power images were obtained using a Nikon Spinning Disk TiE inverted fluorescence confocal microscope attached to an Andor DU-897 EMCCD camera (×100 objective, 8 μm sections). All other images were obtained using the Keyence BZ-X710 microscope as above. The following primary antibodies and probes were used: anti–pro–SP-C (Abcam ab90716), anti-ABCA3 (Abcam ab24751), anti-T1α (podoplanin, Developmental Studies Hybridoma Bank 8.1.1), anti-ezrin (Cell Signaling Technology 3145S), anti–cytokeratin 8 (Origene BP5074), anti–Ki67-FITC (eBioscience 11-5698-90), anti-CD68 (Abcam ab53444 and ab125212), anti-Ager (R&D Systems AF1145), anti–Hopx-647 (Santa Cruz Biotechnology sc-398703), anti-BrdU (MilliporeSigma B2531), anti-GFP (Abcam ab13970), anti–pan-laminin (MilliporeSigma AB2034), anti–integrin β_1_ (MilliporeSigma MAB1997), anti–integrin α_5_ (Abcam ab150361), anti–integrin α_v_ (Abcam ab179475), anti–integrin β_5_ (Invitrogen PA5-118499), anti–integrin β_6_ (Invitrogen PA5-47309), anti–integrin β_8_ (Invitrogen PA5-100843), anti–phospho-p65 (Abcam ab194726), JLA20 (Developmental Studies Hybridoma Bank JLA20-s), and phalloidin (Invitrogen A12380). We applied the following secondary antibodies: anti-rabbit Alexa Fluor 405 (Invitrogen A48258), anti-rabbit Alexa Fluor 488 (Invitrogen A21206), anti-rabbit Alexa Fluor 594 (Invitrogen A21207), anti-rabbit Alexa Fluor 647 (Invitrogen A32795), anti-rat Alexa Fluor 405 (Invitrogen A48268), anti-hamster Alexa Fluor 488 (Invitrogen A21110), anti-goat Alexa Fluor 488 (Invitrogen A11055), anti-goat Alexa Fluor 647 (Invitrogen A21447), anti-chicken Alexa Fluor 488 (Invitrogen A11039), and anti–guinea pig Alexa Fluor 647 (Invitrogen A21450). TUNEL staining was performed on paraffin sections co-immunostained with pro–SP-C, per manufacturer’s kit instructions (Roche 11684795910). RNA in situ hybridization for mouse *Sftpc* was used in conjunction with immunofluorescence staining for the anti-ezrin antibody. RNA in situ hybridization was performed per manufacturer’s instructions (RNAScope, ACDBio), including positive and negative control probes. Quantification of immunostained sections was performed on at least 10 nonoverlapping images obtained with a 40× objective. JLA20 and phalloidin probe detection was quantified on lung sections (10 sections/mouse) imaged with equivalent settings using the corrected total cell fluorescence feature from ImageJ (NIH), which corrects fluorescence-integrated density for the area of the region of interest (pro–SP-C^+^ cells).

### Cell morphometry.

Day 7 and day 21 AT2 cell area and roundness were calculated using the shape descriptor feature of ImageJ with pro–SP-C^+^CD68^–^ cells used to define the region of interest.

### PCLS.

PCLS of 300 μm were generated from the lungs of 3-month-old β_1_^AT2-KO^ and β_1_^fl/fl^ mice, as previously described (56–58). Briefly, slices were cultured in DMEM:F12 for 12 hours, then incubated with BrdU (1 mM, MilliporeSigma B5002) for 4 hours prior to 48 hours of treatment with LPS (MilliporeSigma L2880, 62.5 ng/mL) and/or BAY 11-7082 (100 μM, Tocris, Bio-Techne; Bay11-7821). Slices were then fixed, paraffin-embedded, and sectioned.

### BAL.

After sacrifice, lungs were lavaged with 1 mL sterile PBS. The cells present in lavage fluid were collected by centrifugation (300*g* at 4°C for 10 minutes), resuspended, and counted. Protein content in lavage fluid was measured by BCA protein assay (Pierce catalog 23225) per manufacturer’s instructions.

### Alveolar epithelial cell isolation and G-LISA.

Primary AT2 cells were collected at indicated time points as previously described (8, 9), which yields more than 90% AT2s (59, 60). Briefly, single-cell lung suspension was generated after dispase digestion and serial filtration. Cells were then applied to plates coated with CD32 and CD45 for negative selection. Epithelial cells were then collected from medium after a 2-hour incubation. Cell lysates were then used for G-LISA small GTPase activation assay (Cytoskeleton catalog BK135), where levels of activated RhoA, Cdc42, and Rac1 were detected colorimetrically per manufacturer’s instructions.

### Single-cell data collection.

Sample collection and single-cell sequencing were performed as previously described (61). Briefly, lung lobes were harvested, minced, and incubated for 30 minutes at 37°C in dissociation media (RPMI-1640 from Gibco with 0.7 mg/mL collagenase XI and 30 mg/mL type IV bovine pancreatic DNase, both from MilliporeSigma). After incubation, tissue was disassociated into a single-cell suspension by passage through a wide-bore pipet tip and filtration through a 40 μm filter (Corning 352340). The single-cell lung suspension was then counted, aliquoted, and blocked with CD32 Fc block (BD catalog 553142) for 20 minutes on ice. After a 2% FBS staining buffer wash, cells were incubated with the conjugated primary antibodies anti-CD45 (BD catalog 559864) and anti-Ter119 (BioLegend catalog 116211). Samples from day 21 after LPS were also incubated with anti-CD326 antibody (BD catalog 563477) for epithelial enrichment.

### scRNA-Seq library preparation and next-generation sequencing.

scRNA-Seq libraries were generated using the Chromium Single Cell 5′ library preparation kits (10x Genomics) following the manufacturer’s recommendations and targeting 10,000–20,000 cells per sample. Sequencing was performed on an Illumina NovaSeq 6000. CellRanger Count v3.1 (10x Genomics) was used to align reads onto the mm10 reference genome.

### Analysis of single-cell sequencing data.

Ambient background RNA was cleaned from the scRNA-Seq data with SoupX (62) as described previously (61) using the following genes to estimate the nonexpressing cells, calculate the contamination fraction, and adjust the gene expression counts: *Dcn*, *Bgn*, *Aspn*, *Ecm2*, *Fos*, *Hbbbs*, *Hbbbt*, *Hbaa1*, *Hbaa2*, *Lyz1*, *Lyz2*, *Mgp*, *Postn*, and *Scgb1a1*. For all data sets, quality filtering was then used to remove cells with >15% or <0.1% mitochondrial mRNA and to remove cells with <700 detected genes.

Dimensionality reduction, clustering, and visualization were performed using Seurat v4.0.5 and SCTransform v0.3.2.9008 with glmGamPoi v 1.6.0 (63–65). SCTransform was run with each sequencing run as a batch variable and with the percentage of mitochondrial RNA as a regression variable. Further data cleaning was done to remove gene counts for *Gm42418*, which is likely an rRNA (66). Epithelial cells (i.e., *Epcam^+^* cell clusters) were sorted in silico for downstream analysis. Epithelial cells were annotated with manual inspection of the following marker genes: *Epcam*, *Sftpa1*, *Sftpc*, *Hopx*, *Aqp5*, *Col4a3*, *Ager*, *Foxj1*, *Dynlrb2*, *Mki67*, *Scgb1a1*, *Scgb3a2*, *Cdkn1a*, *Cldn4*, *Ascl1*, and *Scg5*. All charts and heatmaps as part of the scRNA-Seq analysis were generated with ggplot2, and all parts of the analysis were run in R 4.1.1.

Data integration was performed using a previously published data set (12). Reads were downloaded from the National Center for Biotechnology Information’s Gene Expression Omnibus (GSE113049) and processed using CellRanger Count v3.1 with the mm10 genome, followed by dimensionality reduction, clustering, and visualization as described above. Data were integrated with non-SoupX-processed data using the “IntegrateData” function in Seurat, following the workflow for data normalized with SCTransform. Samples from all experiments were combined, and clusters were annotated using marker genes described in the initial publication (12), as well as other canonical marker genes.

Cell label transfer was also utilized with a second published data set (11). The annotated data matrix (h5ad file) was accessed as directed in the analysis code published as part of the methods (11). The raw counts from this annotated data set were then processed using the Seurat workflow described above, while maintaining the published cell annotations. The Seurat “TransferData” function was then used to transfer the cell annotations onto the data set generated here.

### Statistics.

Comparison between 2 groups was performed by 2-tailed *t* test, and 4-way comparison was done by 1- or 2-way ANOVA, as indicated. Individual *P* values, *t* values, *F* values, *df*, and sample size for each group are included in the figure legends and provided in Supplemental Table 1. For graphical data, data represent mean ± SEM. ScRNA-Seq analysis was completed as above.

### Study approval.

All animal experiments were approved by the Institutional Animal Care and Use Committee at Vanderbilt University Medical Center.

### Data availability.

A complete collection of all package versions and code for all steps of the analysis is available at https://github.com/SucreLab/Itgb1LPS (commit ID c52fba2). All sequencing data have been deposited to the NCBI’s Gene Expression Omnibus database with accession number GSE205882. Values for all data points found in graphs are in the Supporting Data Values file. Other data are available upon request.

## Author contributions

EJP conceived the study; performed in vivo experiments, histological analysis, and image analysis; interpreted the data; and wrote the manuscript. JMSS interpreted the scRNA-Seq data and wrote the manuscript. FB, JJG, JTB, and KTF performed histological analysis. PMG performed in vivo experiments, histological analysis, and protein assays. XD and SK performed protein assays. WH and CSJ performed in vivo experiments. JAK, NMN, and YL analyzed the scRNA-Seq data. SHG assisted in manuscript preparation. RZ and TSB conceived of the study, interpreted the data, and wrote the manuscript.

## Supplementary Material

Supplemental data

Supporting data values

## Version 1 06/06/2023

In-Press Preview

## Version 2 07/24/2023

Electronic publication
